# C3G downregulation induces the acquisition of a mesenchymal phenotype that enhances aggressiveness of glioblastoma cells

**DOI:** 10.1038/s41419-021-03631-w

**Published:** 2021-04-06

**Authors:** Sara Manzano, Alvaro Gutierrez-Uzquiza, Paloma Bragado, Celia Sequera, Óscar Herranz, María Rodrigo-Faus, Patricia Jauregui, Stephanie Morgner, Ignacio Rubio, Carmen Guerrero, Almudena Porras

**Affiliations:** 1 Departamento de Bioquímica y Biología Molecular, Facultad de Farmacia, Universidad Complutense de Madrid, Madrid, Spain; 2 Instituto de Investigación Sanitaria del Hospital Clínico San Carlos (IdISSC), Madrid, Spain; 3 Instituto de Biología Molecular y Celular del Cáncer (IBMCC), Universidad de Salamanca-CSIC, Salamanca, Spain; 4 Grupo de Oncología Molecular y Traslacional, Instituto de Investigaciones Biomedicas August Pi i Sunyer (IDIBAPS), 08036 Barcelona, Spain; 5 Department of Anaesthesiology and Intensive Care Medicine, Jena University Hospital, Am Klinikum 1, D-07747 Jena, Germany; 6 Instituto de Investigación Biomédica de Salamanca (IBSAL), Salamanca, Spain; 7 Departamento de Medicina, Universidad de Salamanca, Salamanca, Spain

**Keywords:** CNS cancer, Metastasis

## Abstract

Glioblastoma (GBM) is the most aggressive tumor from the central nervous system (CNS). The current lack of efficient therapies makes essential to find new treatment strategies. C3G, a guanine nucleotide exchange factor for some Ras proteins, plays a dual role in cancer, but its function in GBM remains unknown. Database analyses revealed a reduced C3G mRNA expression in GBM patient samples. C3G protein levels were also decreased in a panel of human GBM cell lines as compared to astrocytes. Based on this, we characterized C3G function in GBM using in vitro and in vivo human GBM models. We report here that C3G downregulation promoted the acquisition of a more mesenchymal phenotype that enhanced the migratory and invasive capacity of GBM cells. This facilitates foci formation in anchorage-dependent and -independent growth assays and the generation of larger tumors in xenografts and chick chorioallantoic membrane (CAM) assays, but with a lower cell density, as proliferation was reduced. Mechanistically, C3G knock-down impairs EGFR signaling by reducing cell surface EGFR through recycling inhibition, while upregulating the activation of several other receptor tyrosine kinases (RTKs) that might promote invasion. In particular, FGF2, likely acting through FGFR1, promoted invasion of C3G-silenced GBM cells. Moreover, ERKs mediate this invasiveness, both in response to FGF2- and serum-induced chemoattraction. In conclusion, our data show the distinct dependency of GBM tumors on C3G for EGF/EGFR signaling versus other RTKs, suggesting that assessing C3G levels may discriminate GBM patient responders to different RTK inhibition protocols. Hence, patients with a low C3G expression might not respond to EGFR inhibitors.

## Introduction

C3G (Crk SH3-domain-binding guanine-nucleotide-releasing factor), encoded by *RAPGEF1* gene, is a guanine nucleotide exchange factor (GEF) for Rap1 and other GTPases from Ras superfamily^1–4^. However, some of its actions are not dependent on its GEF activity^5–7^, being mediated by protein–protein interactions^4,8^. C3G regulates several cellular functions, such as apoptosis, differentiation, and proliferation^4,7,9,10^, being remarkable its role in adhesion and migration^2,11–15^. Accordingly, C3G knock-out mice die before E7.5 due to a defect in integrin-mediated adhesion^2^.

C3G function in human cancer varies with tumor type and stage^4,16^. C3G prevents malignant transformation induced by several oncogenes^5,6,17^. Accordingly, C3G expression is reduced in cervical squamous cell carcinoma^18^. In contrast, C3G is upregulated in non-small-cell lung cancer^19^ and, hepatocarcinoma (HCC), inducing tumor growth^20,21^. High expression of p87C3G isoform is also associated with chronic myeloid leukemia development^22^ and C3G mutations correlate with lymphomas development^23^. In colorectal cancer (CRC), C3G plays a dual role. It promotes tumor growth, while inhibits migration/invasion^13^. C3G also reduces migration in highly invasive breast carcinoma cells^24^. Additionally, C3G is important in the tumor stroma. In particular, C3G promotes the release of pro-angiogenic and pro-metastatic factors from platelets, enhancing tumor growth and metastasis^25^.

C3G can be a mediator of receptor tyrosine kinases (RTKs), such as EGFR^8^, Met^21,26^, insulin receptor^8,27^, TrkA^28^ or ALK^29^. For example, C3G participates in EGF and NGF-induced pathways that promote proliferation and differentiation in neural cells, respectively^28,30,31^.

Even though C3G expression is ubiquitous in humans, C3G levels are higher in brain than in other tissues^1,4,32,33^. C3G regulates important functions in the CNS, such as neural differentiation, neurite outgrowth, and survival^28,30,31,34^. C3G also controls migration and the size of different neuron populations during brain development^11,35–38^. However, little is known about its role in brain tumors. In particular, its function in glioblastoma (GBM) remains unknown.

GBM is considered the most aggressive CNS tumor due to its malignant nature and the lack of effective treatments. CNS tumors are classified by World Health Organization according to the histological and molecular criteria^39,40^. GBMs grade IV astrocytomas, characterized by a high proliferation, low differentiation, high cell heterogeneity, and invasive capacity^39^, are subdivided into IDH-wt (90%) or mutated (10%)^40^. The most frequent genetic alterations in GBMs affect RTK, Rb, and p53 signaling pathways^41,42^. GBMs can also be subdivided into classical, neural, pro-neural, and mesenchymal subtypes^40,43,44^. However, GBM classification remains controversial due to the heterogeneity of tumors.

GBMs invade the surrounding tissue, disseminating through the brain^45^. Epithelial to mesenchymal transition (EMT)-like processes would play a role^46^. High levels of EMT-associated transcription factors, such as *SNAIL1, ZEB1, ZEB2 and TWIST* correlate with a more disseminative and mesenchymal phenotype^46–49^ and can promote stemness^50–53^ and therapy resistance^54^.

GBM aggressiveness hinders its treatment. Moreover, tumor relapse after treatment is very common in GBM patients^44^ and there is no good second-line treatment available. Therefore, more studies are necessary to understand the mechanisms controlling this aggressive pathology in order to find more effective therapies. Given the relevance of C3G in brain development, its key function in adhesion and migration and its role in other cancer types, we hypothesized that C3G could play a relevant role in the tumorigenic and invasive properties of GBM cells. To explore this, we used different human GBM cell models for in vitro and in vivo studies and patient data.

## Results

### C3G is downregulated in glioblastoma, favoring the acquisition of a more invasive phenotype

**Fig. 1:**
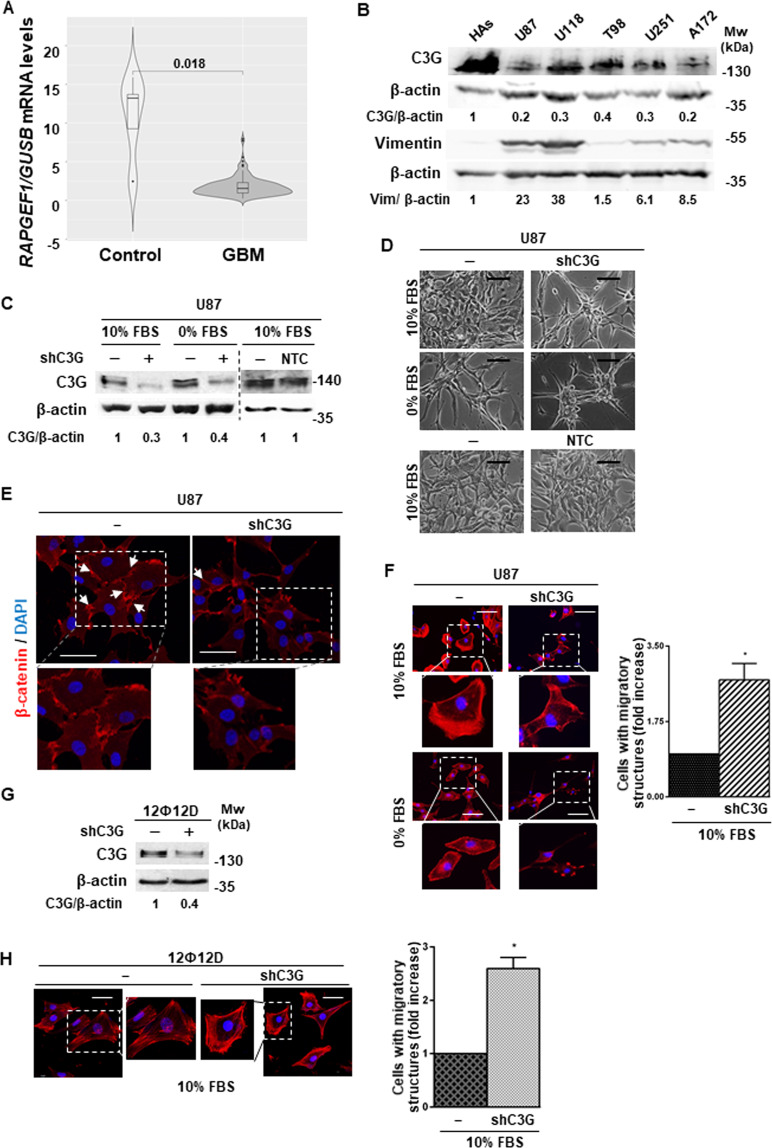
C3G is downregulated in GBM promoting changes in cell morphology. **A** C3G (*RAPGEF1*) mRNA levels in GBM patients and normal brain (171 patients) obtained from TCGA database normalized with *GUSB*. **B** Western-blot analysis of C3G and Vimentin protein levels in the indicated GBM cell lines and human astrocytes (HAs) normalized to β-actin. Densitometric quantification of C3G/β-actin and Vimentin/β-actin ratios are shown. **C**, **G** Western-blot analysis of C3G normalized to β-actin to confirm silencing in U87 and 12Ф12D cells as compared to parental cells and/or cells with control shRNA (NTC). Densitometric quantification of C3G/β-actin ratio is shown. **D** Phase-contrast microscopy images of parental U87, U87shC3G and NTC cells maintained either in the presence (10% FBS) or absence (0% FBS) of serum for 24 h. Scale bars: 50 µm. **E** Representative images of β-catenin (red) and DAPI (blue) staining analyzed by confocal microscopy. Scale bars: 25 µm. **F** and **H** Left panels, immuno-fluorescence microscopy images of phalloidin staining (red) in parental and C3G-silenced U87 and 12Ф12D cells, maintained as indicated. Cell nuclei were stained with DAPI (blue). Scale bars: 50 µm. An amplification of cells inside the square is also shown. Right panels, histograms showing the quantification of the number of cells presenting migratory structures (filopodia, blebs, stress fibers and/or lamellipodia) expressed as fold increase.

The function of C3G in GBM remains unknown. Hence, we first analyzed its expression using databases. A detailed analysis from TCGA-RNA-seq database showed that *RAPGEF1* levels normalized with *GUSB* (Fig. 1A), *ACTB* or *UBC* (Supplementary Fig. 1A) were significantly downregulated in patient GBM tumors. Moreover, *RAPGEF1* downregulation was sex and age independent (Supplementary Fig. 1B), suggesting a putative role for C3G in GBM onset and/or development. In addition, C3G protein levels were decreased in a panel of human GBM cell lines as compared to human astrocytes (Fig. 1B). We also found an inverse correlation between C3G levels and the mesenchymal marker, Vimentin (Fig. 1B), associated with higher aggressiveness in GBM cells^55–57^.

To characterize the role of C3G in GBM, a permanent C3G silencing was performed in a standard GBM U87 cell line using specific shRNAs. C3G protein expression was efficiently down-regulated (60–70%), while non-targeting control (NTC) shRNAs had no effect (Fig. 1C). U87shC3G cells displayed a phenotype with few cell-cell contacts (Figs. 1D and E) and more F-actin migratory structures (mainly, filopodia and blebs) (Fig. 1F). Such morphological changes were not observed in control shRNA cells (Fig. 1D). This was confirmed in 12Ф12D cells, a cell line derived from a patient^58,59^, where C3G knock-down (Fig. 1G) induced similar morphological changes (Supplementary Fig. 2A) and an increase in stress fibers Fig. 1H.

**Fig. 2:**
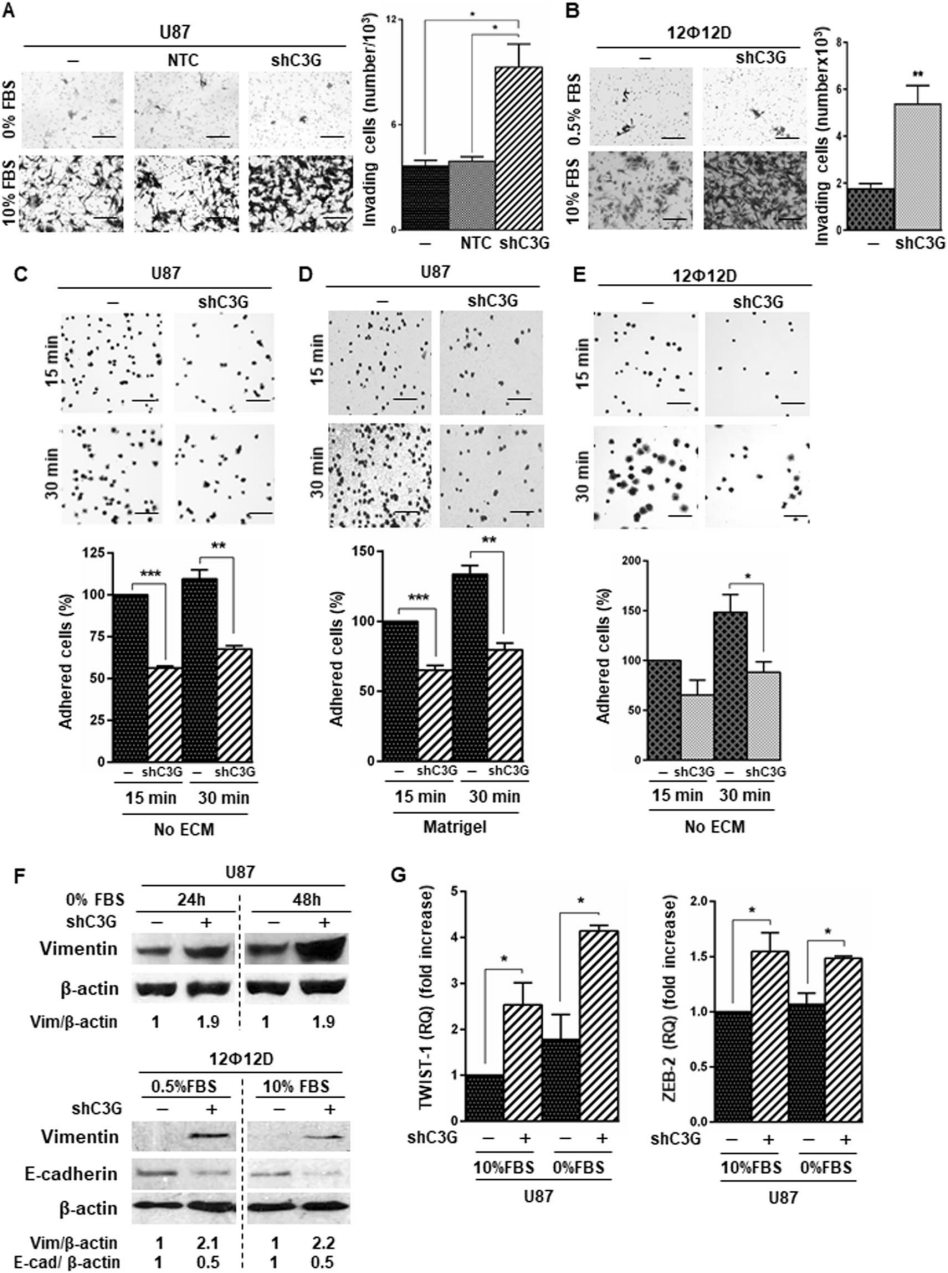
C3G downregulation enhances invasion of GBM cells promoting the expression of mesenchymal markers. Non-silenced or C3G-silenced (shC3G) U87 and 12Ф12D cells, and U87 cells with control shRNA (NTC) have been used. **A** and **B** Invasion through Matrigel of U87 and 12Ф12D cells (respectively) using FBS as chemoattractant (10%). Left panels, representative images of invading cells; right panels, histograms showing the mean value ± S.E.M. of the number of invading cells (*n* = 3). **C**–**E** Adhesion analysis at 15 or 30 min in U87 and 12Ф12D cells. Top panels, phase contrast microscopy representative images of adhered U87 cells on uncoated dishes (no ECM) or coated with Matrigel; lower panels, histograms showing mean value ± S.E.M. of the percentage of adhered cells referred to non-silenced cells at 15 min (*n* = 3). *p ≤ 0.05, **p ≤ 0.01 and ***p ≤ 0.001, compared as indicated. Scale bars (**A**–**E**): 100 µm. **F** Representative western-blot analyses of Vimentin and E-cadherin levels normalized to β-actin in U87 (upper panel) and 12Ф12D cells (lower panel) treated as indicated (*n* = 3). **G** *TWIST1* and *ZEB2* mRNA levels in U87 cells maintained either in the presence (+) or absence (−) of serum. Histograms represent RQ mean value ± S.E.M. referred to parental cells maintained with 10% FBS (*n* = 2–4). *p ≤ 0.05, compared as indicated.

Next, we evaluated the effect of C3G downregulation on GBM migration/invasion and adhesion. We found an enhanced invasiveness in U87shC3G cells as compared to parental or NTC U87cells (Fig. 2A) using serum as chemoattractant. Similarly, C3G silencing increased invasion (Fig. 2B) and migration (Supplementary Fig. 2B) of 12Ф12D cells. Moreover, MMP-2 activity was higher in U87shC3G than in non-silenced cells (Supplementary Fig. 2C). Accordingly, adhesion of U87 and 12Ф12D cells decreased following C3G downregulation (Fig. 2C–E), which would facilitate migration.

To get further insights into the mechanisms used by C3G to regulate invasion in GBM cells, we analyzed EMT markers. C3G silencing increased Vimentin (mesenchymal marker) protein levels in both U87 and 12Ф12D cells and reduced E-cadherin (epithelial marker) levels in 12Ф12D cells (Fig. 2F). In addition, mRNA levels of EMT-associated transcription factors, *TWIST1* and *ZEB2*, were up-regulated in U87shC3G cells (Fig. 2G).

### C3G downregulation alters tumorigenic properties of glioblastoma cells

**Fig. 3:**
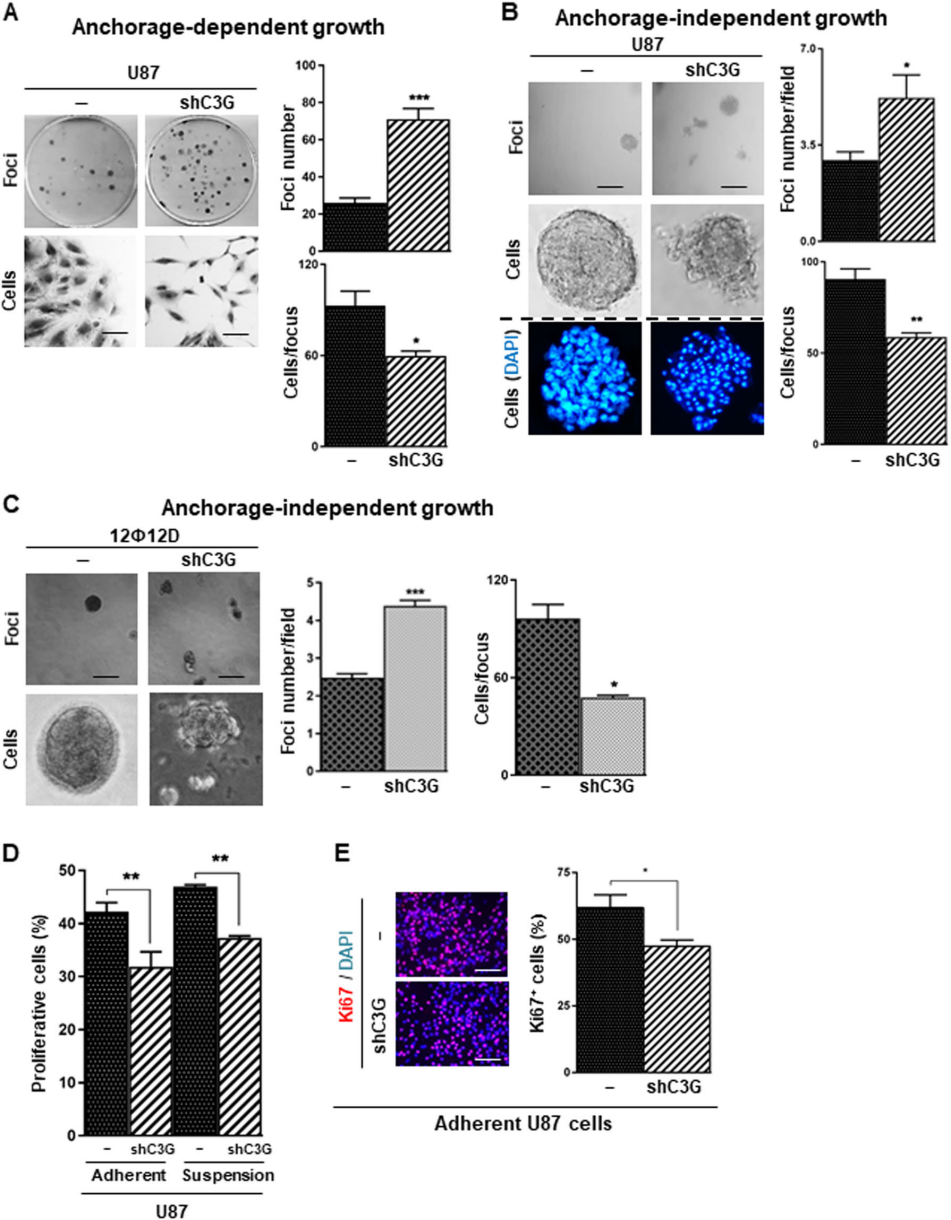
Effect of C3G silencing on tumorigenic and proliferative properties of GBM cells. Non-silenced or C3G-silenced (shC3G) U87 and/or 12Ф12D cells have been used. **A** Anchorage-dependent growth assay in U87 cells. Left panel, representative phase-contrast microscopy images of a macroscopic view of foci (upper images) and an individual focus (lower images); right panel, histogram showing the mean value ± S.E.M. of the total foci number (upper panel) or number of cells per focus (lower panel) (*n* = 5). **B** and **C** Anchorage-independent growth assays in U87 and 12Ф12D cells. Left panels, representative phase-contrast microscopy images of a macroscopic view of foci (upper images), cell organization in an individual focus (middle (**B**) or lower (**C**) images) and nuclei staining with DAPI (*blue*) (B lower panels). Dashed line indicates that DAPI stained nuclei images correspond to an independent experiment. Right panels, histograms showing the mean value ± S.E.M. of the foci number per field (upper) or number of cells per focus (lower) (*n* = 3–4). (**D**) Cell cycle analysis by cytometry of cells maintained in the presence of 10% FBS under adherent or non-adherent conditions (6 h). Histogram represents the percentage of cells in S and G2/M phases of cell cycle ± S.E.M. (*n* = 2–6). *p ≤ 0.05, **p ≤ 0.01, ***p ≤ 0.001, C3G silenced as compared to non-silenced cells under the same experimental condition. (**E**) Ki67 (*red*) staining in adhered U87 cells. Nuclei were stained with DAPI (*blue*). Left panel, representative microscope images; right panel, histogram showing the mean value ± S.E.M. of the percentage of Ki67 positive cells (*n* = 3). *p ≤ 0.05 as compared to non-silenced cells. Scale bars: 100 µm (A, B, C and E).

To determine the role played by C3G in GBM tumorigenic properties, we first performed in vitro functional analyses. Anchorage-dependent and –independent growth assays revealed an increased number of foci upon C3G silencing in U87 cells as compared to parental (Figs. 3A and 3B, respectively) or NTC-U87cells (Supplementary Fig. 3A and 3B). Similar results were obtained using 12Ф12D cells (Supplementary Fig. 3C and Fig. 3C). Interestingly, foci formed by C3G-silenced cells contained fewer and more disseminated cells and cell-cell interactions were reduced, leading to a scattered phenotype (Fig. 3A–C). This suggested that C3G-silenced cells might generate more foci by cell scattering, while being unable to repopulate them. In support of the latest, cell proliferation was decreased in U87shC3G cells, under adherent and non-adherent conditions, according to cell cycle and Ki67 staining analyses (Figs. 3D and 3E). However, no differences were found in apoptosis (data not shown).

**Fig. 4:**
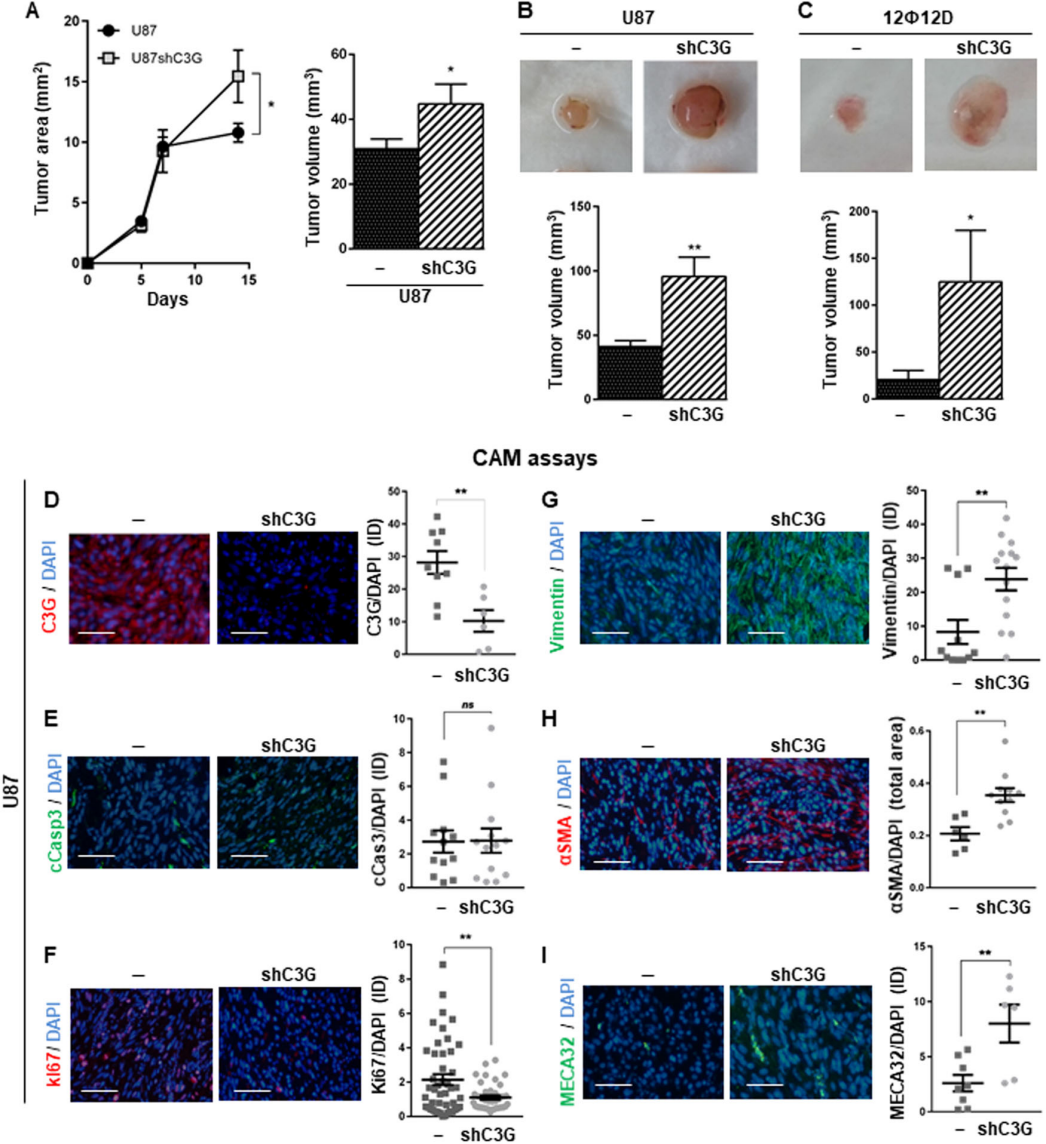
Effect of C3G knock-down on in vivo tumor growth. **A** Xenograft assay using U87 cells. Left panel, graphic showing the mean value of tumor area along the time (0–15 days) ± S.E.M. (two independent experiments, 5 mice/each); right panel, histogram represents the mean value of tumor volume ± S.E.M. at the end-point (15 days). **B** CAM assays using U87 and 12Ф12D cells. Top panels, representative tumors at the end point (7 days); lower panels, histogram represents the mean value of tumor volume ± S.E.M. (three independent experiments, total number of chick embryos 13) at the end point. **D**–**I** Immunofluorescence microscopy analysis in tumors generated by U87 cells in CAM assays of (**D**) C3G (red), **E** cleaved caspase 3 (cCasp3 (green)), **F** Ki67 (green), **G** Vimentin (green), **H** α-Smooth muscle actin (α-SMA (*red*)) and **I** MECA 32 (green). Cell nuclei were stained with DAPI (blue). Left panels, representative images; right panels, graphs represent integrated density mean values normalized with DAPI-positive area ± S.E.M. (*n* = 3). **p ≤ 0.01, compared as indicated. Scale bars: 100 µm.

To evaluate the in vivo function of C3G in GBM tumor growth, xenograft assays were performed. Tumors generated by U87shC3G cells showed a significantly increased size at the end point (Fig. 4A).

To further understand the role played by C3G in GBM tumor growth, we performed CAM assays^60,61^. U87shC3G cells generated larger tumors than non-silenced U87 cells (Fig. 4B) with no apparent necrotic areas (Supplementary Fig. 4A) and less cells/field (Supplementary Fig. 4B and 4C). These results were validated using 12Ф12D cells (Fig. 4C). We confirmed that C3G downregulation was maintained in U87shC3G derived tumors (Fig. 4D). In agreement with in vitro experiments, C3G downregulation had no effect on apoptosis (cleaved caspase-3; Fig. 4E), while reduced proliferation (Ki-67; Fig. 4F) and upregulated Vimentin (Fig. 4G) and the marker of activated fibroblasts (α-SMA; Fig. 4H). Moreover, tumors formed by U87shC3G cells showed a higher density of blood vessels (MECA32; Fig. 4I).

### C3G facilitates activation of EGFR signaling favoring its membrane localization

**Fig. 5:**
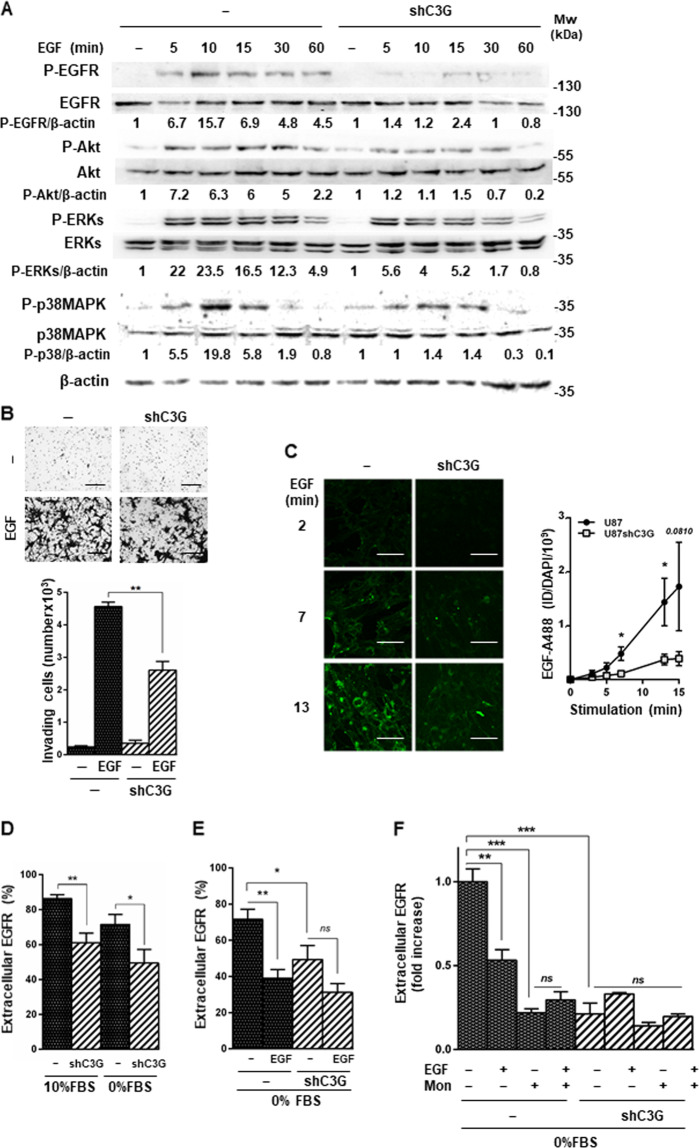
Effect of C3G knock-down on EGFR activity and membrane localization. U87 and U87shC3G cells were used. **A** Representative western-blot analysis of phosphorylated and total levels of EGFR, ERKs, p38MAPK, and Akt proteins normalized to β-actin. Densitometric quantification of these proteins versus β-actin expressed as the fold increase of the value of untreated cells from the corresponding genotype (non-silenced and C3G silenced) (*n* = 3). Untreated shC3G versus non-silenced cells ratios are: P-EGFR/β-actin = 1, P-Akt/β-actin = 2, P-ERKs/β-actin = 1.6, P-p38MAPK/β-actin = 2.6. Serum-starved cells (for 16 h) were stimulated with EGF for 5–60 min or maintained untreated. **B** Invasion through Matrigel in response to EGF. Upper panel, representative phase contrast microscopy images of invading cells; lower panel, histogram showing the mean value ± S.E.M. of the total number of invading cells (*n* = 3). Scale bars: 100 µm. **C** Top panel, representative fluorescence microscopy images of EGFR endocytosis mediated by EGF labeled with Alexa 488 (EGF-A488, *green*) at different time points; lower panel, graph represents fluorescence integrated density (ID) mean values ± S.E.M. (*n* = 5–6). Scale bars: 50 µm. **D** and **E** Flow cytometric analysis of cell surface EGFR levels. **D** Cells were maintained in the presence (10% FBS) or absence of serum (0% FBS). **E** Cells were treated with EGF for 2 h to induce endocytosis or maintained untreated. Histograms show the percentage of cells presenting EGFR in the membrane (mean value ± S.E.M. (*n* = 6)). *p ≤ 0.05 and **p ≤ 0.01, compared as indicated. **F** Effect of recycling inhibition on cell surface EGFR levels analyzed by flow cytometry. Cells were maintained untreated or treated with monensin (Mon) (10 μM, 1 h), EGF (2 h) or both (EGF + Mon). Histogram showing the mean value ± S.E.M. of the percentage of cells presenting EGFR in the membrane (*n* = 3). **p ≤ 0.01 and ***p ≤ 0.001, compared as indicated.

Signaling by RTKs is broadly implicated in driving GBM onset and development. EGFR alterations and/or overexpression are frequent in GBM patient tumors^62^. Therefore, we analyzed if C3G regulated EGFR signaling in GBM cells. C3G silencing reduced ligand-induced EGFR phosphorylation in U87 cells, but not total EGFR levels (Fig. 5A). In addition, p38MAPK, ERKs, and Akt phosphorylation levels decreased in response to EGF in C3G-silenced cells (Fig. 5A). Next, we studied the functional consequences of the defective EGF/EGFR signaling in GBM cells with C3G downregulation. We observed a significant decrease in EGF-induced invasion upon C3G silencing in the absence of chemoattractant (Fig. 5B).

C3G controls the exocytosis of angiogenic factors by platelets through interaction with VAMP7^25^, a protein involved in vesicle trafficking^63^. Therefore, we studied if the alterations in EGFR signaling and functionality could be a consequence of changes in receptor endocytosis and/or recycling. We detected a reduction in activation-induced EGFR internalization in C3G knock-down cells (Fig. 5C). This suggested that C3G is involved in EGF-mediated EGFR endocytosis and/or recycling. Using flow cytometry as a complementary approach, we found that EGFR levels on cell surface were higher in parental U87 than in U87shC3G cells, both in the presence and absence of serum (Fig. 5D). As a positive control of EGFR endocytosis, serum-deprived cells were treated with EGF for 2 h. In parental U87 cells, the presence of EGFR in the membrane was highly reduced upon EGF treatment (Fig. 5E), according to the rapid internalization observed by microscopy (Fig. 5C). However, in U87shC3G cells the low levels of EGFR at cell surface remained unchanged in response to EGF (Fig. 5E). Moreover, inhibition of recycling by monensin^64^ treatment significantly reduced EGFR levels at cell surface in non-silenced cells, while it had no effect on C3G-silenced cells (Fig. 5F). These findings indicate that C3G would be required for EGFR membrane localization in GBM cells, mainly by favoring receptor recycling.

### C3G downregulation enhances the activation of several tyrosine kinase receptors in glioblastoma cells

**Fig. 6:**
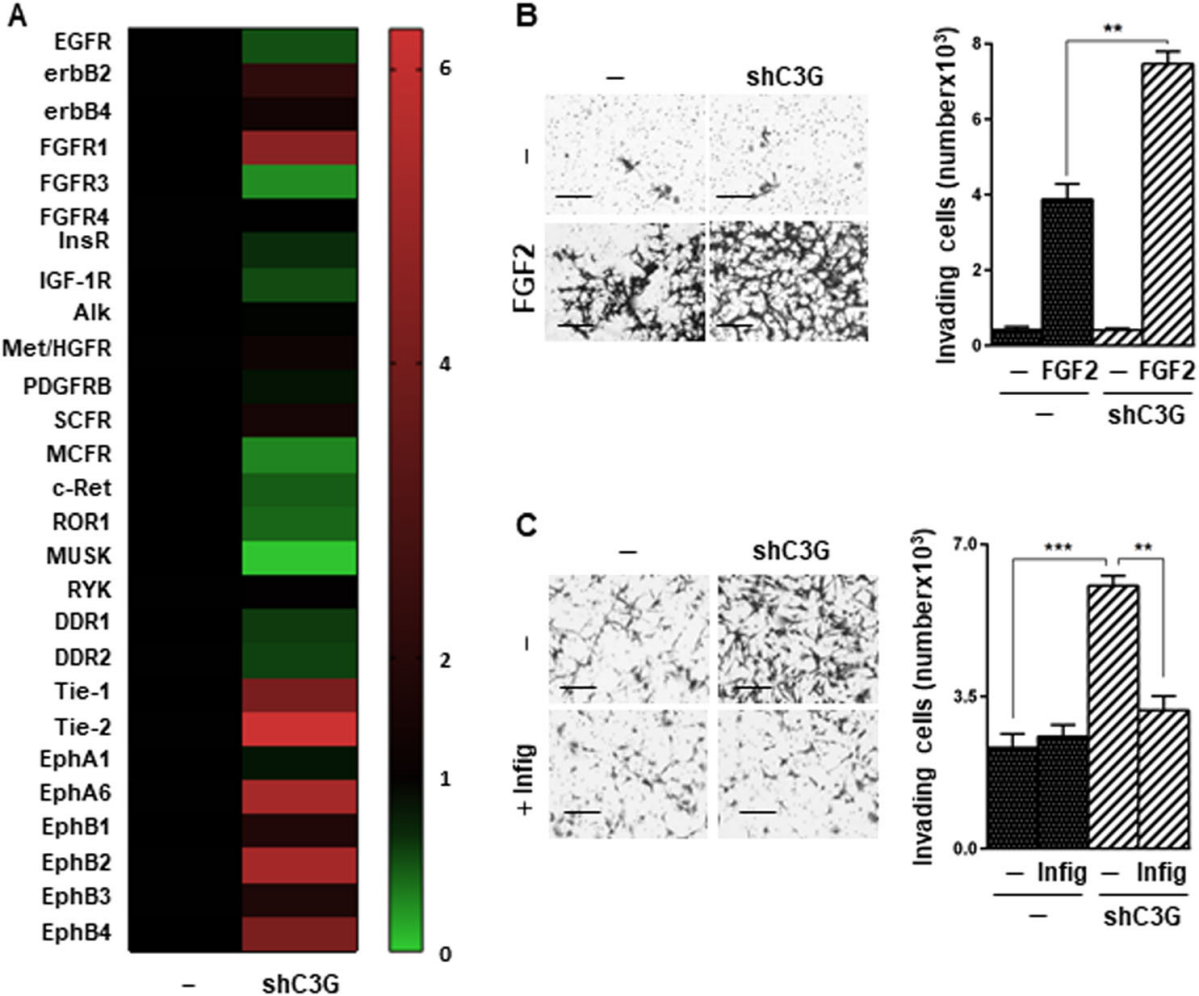
C3G downregulation increases the phosphorylation of FGFR1 and other RTKs, promoting cell invasion. **A** Heatmap represents the relative phosphorylation levels of 27 RTKs present in the proteome profiler human phospho-RTK array. For each receptor, the mean value of the densitometric quantification of C3G silenced cells values as compared to parental cells (*n* = 2). **B** FGF2-induced invasion. Cells seeded in the upper chamber of transwells were maintained in the absence of serum and were treated with FGF2. Left panel, representative phase contrast microscopy images of invading cells; right panel, histogram showing the mean value ± S.E.M. of the total number of invading cells (*n* = 3). *p ≤ 0.05 and **p ≤ 0.01, compared as indicated. Scale bars: 100 µm. **C** Effect of infigratinib on invasion using serum (10%) as chemoattractant. Left panels, representative phase contrast microscopy images of invading cells; right panel, histogram showing the mean value ± S.E.M. of the total number of invading cells (*n* = 3). **p ≤ 0.01 and ***p ≤ 0.001, compared as indicated. Scale bars: 100 µm.

Although invasion was enhanced in U87shC3G cells using serum as chemoattractant, EGF driven invasion was decreased. Therefore, we evaluated the phosphorylation of RTKs using a proteome profiler human phospho-RTK array, searching for potential mediators of the increased invasion induced by C3G downregulation in response to serum. This analysis revealed multiple changes induced by C3G knock-down in U87 cells (Fig. 6A and Supplementary Fig. 5). Among them, we found a significant upregulation in the phosphorylation of FGFR1, erbB2, Eph family members (e.g., EphA6, EphB1, EphB2, and EphB4) and Tie1/2. Several of these RTKs have been associated with cell migration and invasiveness.

Taking into account the relevance of FGFR1 in GBM^65^, we evaluated the effect of its ligand, FGF2, on invasion. U87shC3G cells stimulated with FGF2 showed higher invasiveness than parental cells (Fig. 6B). Moreover, when serum was used as chemoattractant, invasion was prevented by infigratinib, a FGFR1/2/3 inhibitor (Fig. 6C). Altogether, these results indicate that C3G differentially regulates the localization and functionality of selected RTKs in GBM cells, decreasing EGFR activation, while increasing FGFR1 and other RTKs activation. This results in an overall enhanced invasiveness, in part mediated by FGFR1.

### ERKs are involved in the pro-invasive effect of C3G downregulation in glioblastoma cells

**Fig. 7:**
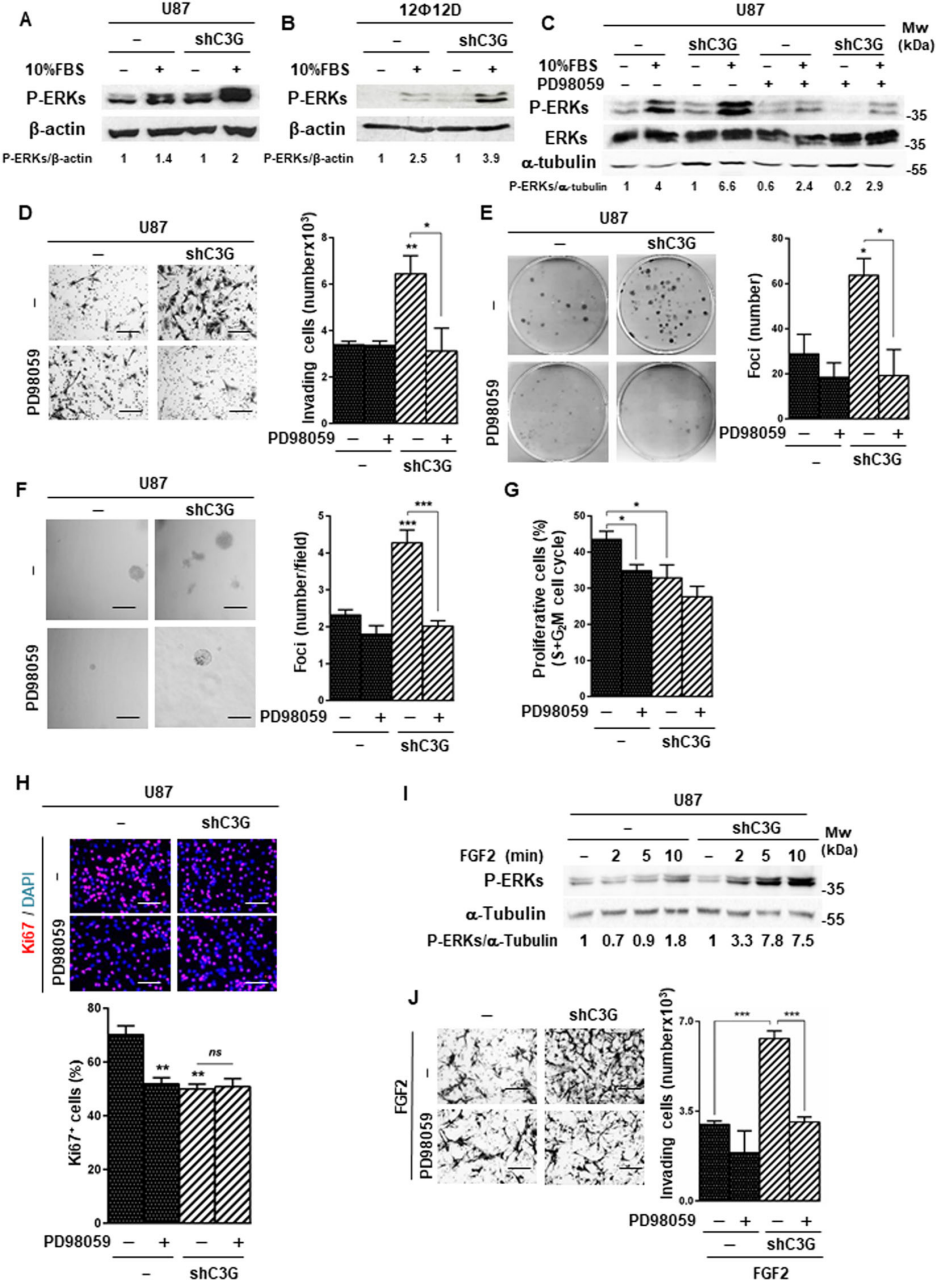
C3G downregulation enhances ERKs activation in response to serum and FGF2, promoting invasion. **A**–**C** Representative western-blot analysis of phosphorylated ERKs levels normalized with β-actin in U87/U87shC3G cells (**A** and **C**) and 12Ф12D/12Ф12DshC3G cells. **B** Serum-deprived cells were maintained untreated (−) or treated (+) with 10% FBS (FBS) for 10 min, either in the presence or absence of PD98059, as indicated. Densitometric analysis of P-ERKs/β-actin (**A**, **B**) or P-ERKs/α-Tubulin (**C**) ratio expressed as the fold increase of the value of untreated cells from the corresponding genotype (non-silenced and C3G silenced) (*n* = 3). **D** Invasion through Matrigel using 10% FBS as chemoattractant. Left panel, representative phase contrast microscopy images of invading cells untreated or treated with PD98059; right panel, histogram showing the mean value ± S.E.M. of the number of invading cells (*n* = 3). *p ≤ 0.05, **p ≤ 0.01, C3G-silenced versus non-silenced cells or compared as indicated. **E** Anchorage-dependent growth assay in the presence or absence of PD98059. Left panel, representative macroscopic images of foci; right panel, histogram showing the mean value ± S.E.M. of foci number (*n* = 3). *p ≤ 0.05, C3G silenced versus non-silenced cells or compared as indicated. **F** Anchorage-independent growth assay in cells maintained untreated or treated with PD98059. Left panel, representative images of a microscopic view of foci; right panel, histogram showing the mean value ± S.E.M. of the total foci number per field (*n* = 4). ***p ≤ 0.001, C3G silenced versus non-silenced cells or compared as indicated. **G** Cell cycle analysis by flow cytometry. Histogram represents the percentage of cells in S and G2/M phases of cell cycle ± S.E.M. (*n* = 3). *p ≤ 0.05, C3G silenced versus non-silenced cells or compared as indicated. **H** Immunofluorescence analysis of Ki67 (red)/DAPI (blue) staining. Upper panel, representative images; lower panel, histogram showing the percentage of Ki67 positive cells (mean value ± S.E.M.). **I** Representative western-blot analysis of P-ERKs levels in response to FGF2 stimulation (5–10 min) normalized to β-actin. Densitometric analysis of P-ERKs/α-tubulin ratio expressed as the fold increase of the value of untreated cells from the corresponding genotype (non-silenced and C3G silenced) (*n* = 3). **J** Invasion through Matrigel in response to FGF2 in the absence and presence of PD98059. ***p ≤ 0.001, compared as indicated. Scale bars: 100 µm.

C3G regulates several intracellular signaling pathways involved in migration and invasion^12,13^. In particular, C3G downregulation in MEFs and HCT116 colon carcinoma cells enhanced ERKs activation^7,13^. In GBM cells, EGF-induced ERKs phosphorylation decreased upon C3G downregulation. However, in response to serum, C3G silencing upregulated phospho-ERKs levels in U87 (Fig. 7A) and 12Ф12D (Fig. 7B) cells.

To assess if C3G effects were mediated by ERKs, we inhibited this pathway with PD98059^66^. PD98059 treatment, which decreased P-ERKs levels in parental and U87shC3G cells (Fig. 7C), significantly reduced serum-induced invasion of U87shC3G cells to the levels of parental cells (Fig. 7D) and migration of 12Ф12DshC3G cells (Supplementary Fig. 6). The effect of C3G downregulation on anchorage-dependent (Fig. 7E) and –independent (Fig. 7F) growth was also prevented by ERKs inhibition. Moreover, cell proliferation in non-silenced U87 cells was reduced by PD98059 (Fig. 7G and 7H), having no effect on C3G-silenced cells.

As FGF2-induced invasion was enhanced in U87shC3G cells, we analyzed ERKs activation in response to FGF2 and found increased P-ERKs levels (Fig. 7I). Moreover, ERKs inhibition prevented FGF2-induced invasion (Fig. 7J). Therefore, the increased FGFR1 activation upon C3G downregulation in GBM cells might enhance invasion by upregulating ERKs activation.

## Discussion

C3G regulates relevant processes for tumor progression including proliferation, apoptosis, adhesion, invasion and angiogenesis^7,12,13^. However, C3G function in cancer appears to depend on the context^4,16^. In this study, we uncover a novel key function for C3G in GBM. Public databases revealed that C3G (*RAPGEF1)* mRNA levels are downregulated in GBM patient samples. C3G protein expression is also reduced in human GBM cell lines, suggesting that C3G levels could be downregulated during GBM onset and/or progression.

We demonstrate that C3G downregulation enhanced migration/invasion of GBM cells (U87 and 12Ф12D). These data are in agreement with the increased migratory properties of C3G deficient MEFs, C3G knock-down CRC and HCC cells^13,21^ and with the inhibitory effect of C3G overexpression on the migration of breast carcinoma cells^24^. In CRC HCT116 cell line, C3G downregulation induces actin cytoskeleton reorganization and increases MMP2/9 activities through upregulating p38αMAPK activity^13^. In GBM cells with C3G downregulation, the enhanced migration/invasion might be a consequence of an EMT-like process that promotes the acquisition of a more mesenchymal phenotype, supported by increased Vimentin levels, MMP2 activity, and *TWIST1* and *ZEB2* mRNA levels^67^. Although the role of EMT in GBM is controversial due to brain plasticity, its non-epithelial characteristics, similarity with astrogliosis and the mesenchymal profile of most GBMs^46,68^, it is accepted that GBM cells can acquire a more mesenchymal phenotype^55–57^, associated with high Vimentin levels, invasiveness, and poor prognosis.

Previously, we reported that C3G downregulation in CRC and HCC cells reduced tumor growth and size^13,21^. However, C3G-silenced GBM cells form bigger tumors in xenograft and CAM assays, even though proliferation is decreased both in vitro and in vivo. This correlates with the higher number of foci with less cells/focus formed by C3G knock-down GBM cells in anchorage-dependent and -independent growth assays and the lower number of tumor cells in CAM-derived tumors. Therefore, this lower cell density, loss of cell-cell interactions, and higher mobility of cells with C3G downregulation lead to larger tumors. Moreover, tumors originated by C3G-silenced cells in CAM assays present higher levels of α-SMA and MECA32, pointing to enrichment in stroma and blood vessels, most likely by infiltration of host-niche cells. Such a scenario could contribute to increase the size of tumors with C3G downregulation. Although cellularity usually correlates with a higher aggressiveness and poor prognosis, the invasive capacity of GBM cells is also associated with treatment resistance, recurrence and poor overall survival^69^. Therefore, C3G downregulation in GBM cells would induce a higher aggressiveness.

Our data also unveil a novel function of C3G controlling the signaling elicited by RTKs, which represent the most commonly altered molecules in glioma^70^. C3G downregulation in GBM cells reduces the amount of EGFR at the cell surface. This reflects defects in receptor recycling and would explain the defective EGFR-mediated signaling and pro-invasive effect. Although the precise mechanisms involved need to be characterized in detail, the regulation of actin cytoskeleton organization by C3G might play a role in the proper recycling and membrane localization of EGFR^71^. C3G controls exocytosis of angiogenic factors in platelets through interaction with VAMP7^25^, which is involved in the secretory pathway that allows EGFR localization in membrane microdomains, also regulating EGFR endocytosis and signaling^72^. Moreover, other SNAREs collaborate with VAMP7 and other VAMPs in cellular vesicle trafficking, including that of EGFR to the membrane^73^. Therefore, C3G might favor EGFR membrane localization and recycling, acting through VAMP proteins. However, other mechanisms might contribute to the defective EGF/EGFR signaling, such as the reduced formation of EGFR-Crk-C3G complexes^28^.

Based on the increased phosphorylation of several, but not all, RTKs in C3G-silenced GBM cells, C3G seems to differentially regulate signals mediated by distinct RTKs, leading to an overall pro-invasive phenotype when C3G levels are low. For example, we observed a strong up-regulation of FGFR1 phosphorylation and FGF2-mediated ERKs activation in GBM cells with C3G downregulation, leading to increased invasiveness. This is in agreement with FGFR1-induced expression of EMT-associated genes in GBM^65^. Moreover, the effect of C3G silencing resembles the pro-invasive action of Abl downregulation or inhibition, but not its anti-tumorigenic effect^74^. This could reflect the differential impact of Abl *versus* C3G on the activity or expression of individual RTKs.

Our data also revealed a remarkable increase in the levels of phosphorylated EphB2 in GBM cells with C3G downregulation, which promotes migration and invasion of GBM and GBM cancer stem cells^75,76^. Therefore, EphB2 might contribute to favor invasion of C3G knock-down GBM cells. Similarly, the up-regulation of ErbB2 phosphorylation found in C3G-silenced cells might also facilitate migration^77^ and could contribute to induce resistance to EGFR inhibition therapy^78^.

Tie2 and Tie1 phosphorylation is also upregulated in C3G-silenced GBM cells, which suggests that C3G downregulation might facilitate an endothelial transdifferentiation, a process occurring in GBM, associated to therapy resistance^79,80^.

Finally, the upregulation of ERKs activation in response to serum and FGF2 in C3G silenced cells might be responsible of enhancing invasiveness of GBM cells, as ERKs inhibition prevents this effect. Moreover, the upregulation of the activity of several RTKs, prominently FGFR1, detected in the array might be responsible for this high ERKs activity.

**Fig. 8:**
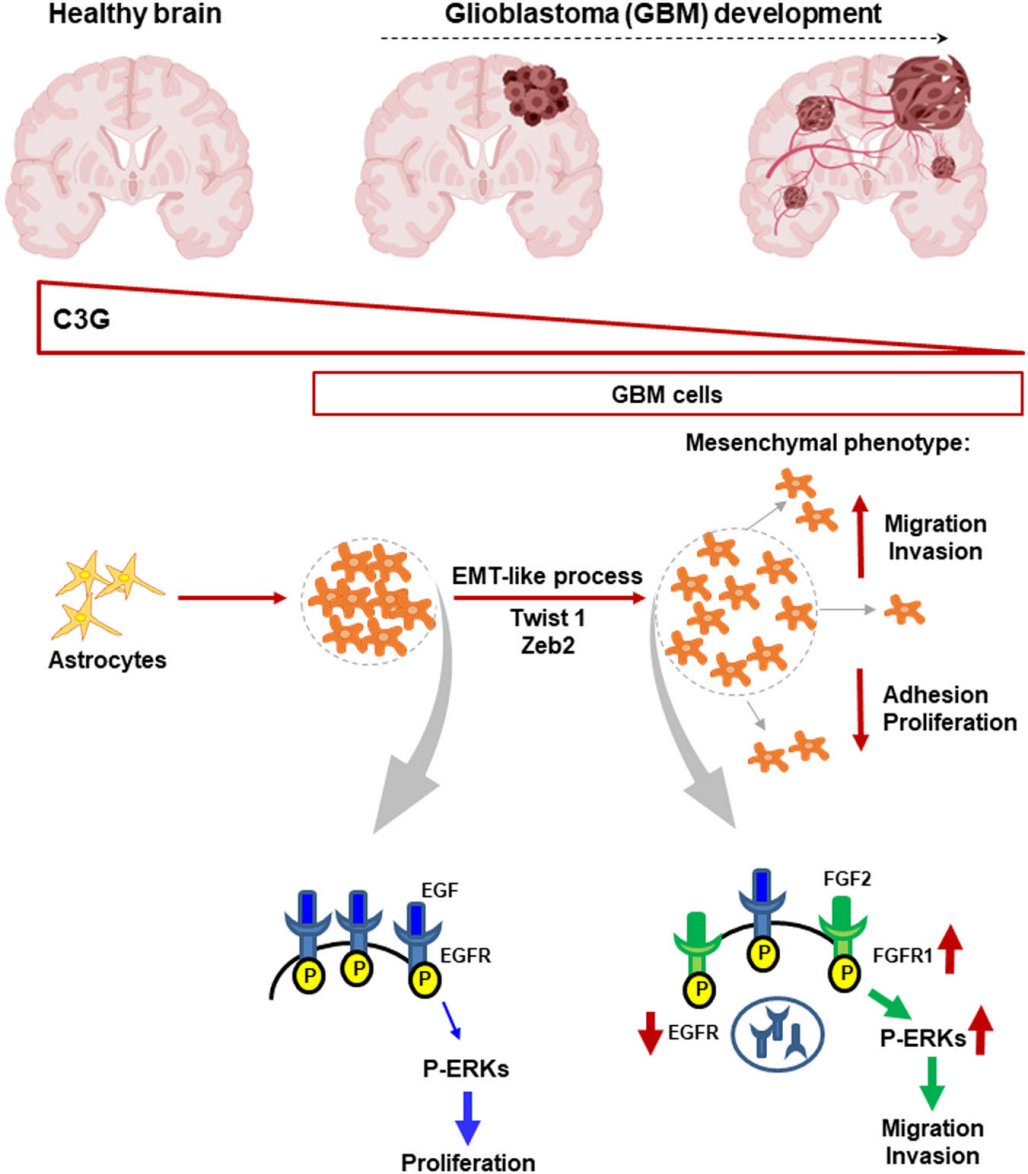
Graphical abstract showing the function of C3G in glioblastoma. C3G levels are high in healthy brain and decrease in glioblastoma (GBM) cells and patient samples. This C3G downregulation enhances migration and invasion of GBM cells by mechanisms that depend on hyperactivation of RTKs such as FGFR1 acting through ERKs. In contrast, EGFR activity is down-regulated due to its low presence at the cell surface.

In summary, we uncover C3G as a novel key player in GBM biology and tumor progression (Fig. 8). C3G is down-regulated in GBM, promoting the acquisition of a more mesenchymal and invasive phenotype, giving rise to larger tumors with less proliferation, but more stromal cells and vessels. Furthermore, the defective EGFR signaling might contribute to resistance to anti-EGFR therapy in patients with low levels of C3G. In contrast, other RTKs and ERKs might represent alternative therapeutic targets in these patients based on the upregulation of their activities. Future studies would allow to further characterize C3G function in RTKs regulation, which may be also of value for designing novel and personalized therapeutic approaches.

## Materials and methods

### Analysis of RAPGEF1 expression in glioblastoma patients using public genomic databases

C3G (*RAPGEF1)* mRNA-seq normalized expression in GBM patients was analyzed using 171 samples from TCGA (The Cancer Genome Atlas), grouped into control and GBM tumor. mRNA-seq and clinical data of TCGA GBM patients was downloaded from FIREBROWSE (http://firebrowse.org/).

### Cell culture and treatments

The human glioblastoma U87MG (from ATCC) and the non-commercial GBM 12Ф12 cell line with a stem-like phenotype (mycoplasma free), derived from a patient GBM tumor^58,59^, were used. Cells were grown in DMEM supplemented with 10% fetal bovine serum (FBS). This induces the differentiation of 12Ф12 cells (12Φ12D cells).

C3G was stably knocked-down using a mixture of human C3G shRNAs^13^ (Santa Cruz Biotechnology, sc-29863-V). As a control, non-targeting shRNAs (Santa Cruz Biotechnology sc-108080) were used. Cells were selected with puromycin (1 µg/ml) (Panreac Applichem, A2856), generating a pool of clones.

Human astrocytes were grown in astrocyte medium (ScienCell#1801) with 10% FBS (ScienCell#0010) and astrocyte growth supplement (ScienceCell#1852).

For different assays, cells were stimulated with EGF (10 ng/ml; R&D 236-EG-200), FGF2 (50 ng/ml; Preprotech AF-100-18B) or 10% FBS. ERKs were inhibited by PD98059 (20 µM; Calbiochem#513000) and FGFR1 signaling by infigratinib (1 μM).

### Western blot analysis

Protein extracts and western blot analysis were carried out as described^13^. Membranes were probed with primary antibodies at 1:1000 dilution or as indicated: C3G H-300 (SCBsc-15359), C3G Home-designed for N-terminal domain (Genosphere, 1:500), Vimentin (BD#550513), E-cadherin (BD#610182), P-Y1068-EGFR (CST #3777), EGFR (CST #4267), P-Thr180/Y182-p38MAPK (CST#9211), p38α MAPK (SCB#9211), P-Thr202/Y204-ERKs (CST#9101), ERKs (CST#9102), P-Ser473-Akt (CST#9271), Akt (CST#9272), β-actin (CST#3700, 1:2500) and α-Tubulin (CST#3873S, 1:2500).

### RNA extraction and RT-qPCR analysis

Total RNA isolation and RT-qPCR analysis was performed as described^21^. Total RNA was isolated using NucleoSpin RNA kit (Macherey-Nagel# 740955.50) and reverse transcribed using SuperScript III RT kit (Invitrogen). cDNA was amplified using specific primers for *TWIST1, ZEB2* and *GUSB* to normalize and detected by SYBR Green (Roche 04913850001) using 7900 Fast Real Time System (Life Technologies 4329001). Ct (threshold cycle) for a gene minus Ct for GUSB = ΔCt and then, referred to non-silenced control values (sample ΔCt-non-silenced ΔCt = ΔΔCt) to calculate RQ (2^−ΔΔCt^).

### Analysis of F-actin organization

Cells seeded on 2% gelatin-coated glass coverslips were fixed with 4% paraformaldehyde (PFA) for 20 min. F-actin was stained with rhodamine-conjugated phalloidin (Sigma Aldrich-P1951) as described^22^. Samples were visualized in a Leica TCS-SL confocal microscope.

### EGFR endocytosis analysis

Cells seeded on 2% gelatin-coated glass-bottomed cell culture dishes were incubated for 30 min in phenol-red-free DMEM medium supplemented with 0.1% BSA. Cell dishes were placed into a LSM510 confocal microscope equipped with a thermostated chamber. Cells were stimulated on-stage with Alexa-Fluor488-labeled EGF (50 ng/ml; Invitrogen-E13345) and imaged alive at different time points.

### Adhesion assay

Cells were seeded on either Matrigel (5μg/cm^2^)-coated or uncoated plates in a medium containing 10% FBS. Adhered cells at 15 min were fixed, stained with crystal violet and counted using Eclipse TE300 Nikon microscope.

### Invasion assay

Invasion was assayed in Matrigel (333 µg/cm^2^; Corning#356234) coated transwells (BD#353097). 25000 cells were seeded in the upper chamber in serum-free medium. 10% FBS-medium, placed in the lower chamber, acts as chemoattractant. After 24 h, cells from the lower chamber were fixed with 4% PFA, stained with crystal violet and counted using an Eclipse TE300 Nikon microscope. To evaluate the effect of EGF, FGF-2 or PD98059 on invasion, cells in the upper chamber were treated with these compounds and no serum was added into the lower chamber.

### Cell cycle analysis

Cells in the medium and trypsinized cells were centrifuged at 2500 rpm 5 min at 4 °C, fixed with cold ethanol (70%) and washed with PBS. Cells resuspended in PBS were incubated 30 min with RNase (0.1 µg/ml) at 37° C. Propidium iodide (0.05 µg/µl) was added and cell cycle analyzed by flow cytometry. Cells in suspension (6 h) were directly pelleted and processed with the same protocol.

### Analysis of cell surface EGFR and its recycling

Cells were detached using Ca^2+^/Mg^2+^-free PBS, resuspended in PBS and incubated with EGFR affibody conjugated with FITC (Abcam#ab81872; 1:200) for 30 min. FITC intensity was measured in an Accuri BD FACS Flow Cytometer. To evaluate recycling, a monensin^64^ (10 μM) 1 h pretreatment was performed. Fluorescence intensity was measured using ImageJ Software and was expressed as integrated density (ID): *i*ntensity mean value of the image multiplied by the positive area percentage.

### Wound healing and anchorage-dependent and -independent growth assays

Wound healing and anchorage-dependent and -independent growth assays were performed as previously described^21,22^. For anchorage-dependent growth, cells (300/60 mm plates) were seeded in a medium supplemented with 10%FBS. To measure anchorage-independent growth, cells (3000/24 multiwell plates) resuspended in 0.35% agar (Sigma Aldrich #A9414) were poured onto a layer of 0.5% agar (in complete medium). Medium was renewed every 3 days. After 14 days, colonies were stained with 0.005% crystal violet.

### Xenograft assay

U87 and U87shC3G cells were subcutaneously injected (6 × 10^6^) into the flanks of Athymic Nude-Foxn1nu female mice (Envigo#6903 F; *n* = 5/condition). Tumor growth was monitored up to 15 days. Then, tumors were excised, measured and fixed in 4% PFA. Tumor size was calculated by the formula (4/3)×π(r1)×(r1)×(r2), where r1 is the longest and r2 the shortest radius. All animal experiments were carried out in compliance with the European Community Council Directive (2010/63/EU), following guidelines for animal research approved by Comunidad Madrid (Spain) with reference PROEX028/17.

### Chick chorioallantoic membrane assays

Chick chorioallantoic membrane (CAM) assays were performed as previously described^60,61^ using premium specific pathogen-free, 9-day-old embryonated chicken eggs (Gibert farmers). U87/U87shC3G (1 × 10^6^) or 12Ф12D/12Ф12DshC3G (2 × 10^6^) cells diluted in PBS with Matrigel were inoculated. After 7 days, tumors were excised, measured and fixed with 4% PFA. Tumor size was calculated as indicated above.

### Analysis of paraffin-embedded tumor samples

Tumors from CAM assays were embedded in paraffin and cut into 8 µm sections and processed as previously described^13^. After deparaffinization and rehydration, antigen retrieval with citrate buffer (10 mM, pH = 6) was performed^3^. Samples were, then, permeabilized with 0.3% Triton X-100, blocked with 1% goat serum-3% BSA-PBS (30 min at RT) and incubated overnight at 4 °C with primary antibodies diluted in blocking solution: C3G (Bethyl, A301-965A; 1/50), cleaved-Caspase 3 (CST, #9664S; 1/100), Ki67 (CST, # 9449; 1/100), Vimentin (BD, #550513; 1/100), α-SMA (Dako, #M0851; 1/50) and MECA32 (Pharmingen, #550563; 1/50). After washing, sections were incubated with secondary antibodies (goat anti-mouse-Alexa 555 or goat anti-rabbit-Alexa 555, 1/200) and DAPI for 2 h at RT in dark. Finally, sections were mounted with mowiol (Thermo Fisher, P36930).

### Immunofluorescence analysis of β-catenin and Ki67

Cells were seeded on glass coverslips pre-coated with 2% gelatin (Sigma#G9391) and maintained 24 h in culture medium supplemented with 10% FBS. Cells were washed with PBS twice, fixed with 4% PFA (20 min), washed with PBS and permeabilized with PBS-0.1% Triton X-100-0.5% BSA (20 -min at RT). Then, cells were incubated with blocking solution (5% BSA-1.5% goat serum in PBS) for 1 h at RT. Next, cells were incubated with anti-β-catenin (BD#610154) or anti-Ki67 (Abcam#ab15580) antibodies (1:50) in blocking solution, overnight at 4°C. Then, cells were washed with PBS and incubated with secondary antibodies (goat anti-mouse-Alexa 555 or goat anti-rabbit-Alexa 555, respectively, 1:250) and DAPI (Panreac#-A4099) in blocking solution. After washing with PBS, coverslips were mounted with mowiol. Images were captured using a Nikon Eclipse TE300 microscope coupled to a camera. For Ki67 quantification, the percentage of positive nuclei/field was determined using ImageJ software.

### RTK phosphorylation analysis

To evaluate phosphorylation of several RTKs, serum-starved cells (60–70% confluence) treated with 10% FBS for 4 h were washed with PBS and lysed using lysis buffer 17 from kit, supplemented with aprotinin (10 µg/ml) and leupeptin (10 µg/ml) and analyzed with human phospho-RTK array Kit (R&D#ARY001B) following the manufacturer’s protocol. Once membranes were blocked with buffer 2, they were incubated overnight at 4 °C with lysates (300 µg proteins) diluted in array buffer 1, washed and incubated for 2 h at RT with anti-phospho-tyrosine-HRP antibody. Then, after washing, membranes were incubated with the kit Chemi Reagent and visualized with ‘VWR Imager Chemi Premium’ documentation system.

### Statistical analysis

Data are represented as the mean values ± S.E.M (*n* ≥ 3) of independent experiments. Unpaired Student’s *t*-test was used for comparison of two experimental groups and one-way or two-way ANOVA analyses to compare more than two groups with one or two variables using GraphPad Prism 7.0 software. Differences were considered significant when p value was *p* ≤ 0.05.

## Supplementary information

Spplementary material and figures
