# Supplementary material for: C3G downregulation induces the acquisition of a mesenchymal phenotype that enhances aggressiveness of glioblastoma cells
Source: Cell Death Dis. 2021 Apr 6;12(4):348. doi: 10.1038/s41419-021-03631-w (PMC8024353; doi:10.1038/s41419-021-03631-w)
Supplement: Supplementary file 1 — Spplementary material and figures [file 41419_2021_3631_MOESM1_ESM.pdf]

Supplementary information

Primers used for RT-qPCR analysis

Primers used for: *TWIST1* f-CAAAGAAACAGGGCGTGGGG and r-CAGAGGTGTGAGGATGGTGCC; *ZEB2* f-AATGCACAGAGTGTGGCAAGGC and r-ATCTGGCGTTCCAGGGACTCAT; *GUSB* f-ATCACCGTCACCACCAGCGT and r-GTCCCATTCGCCACGACTTTG.

Supplementary figures

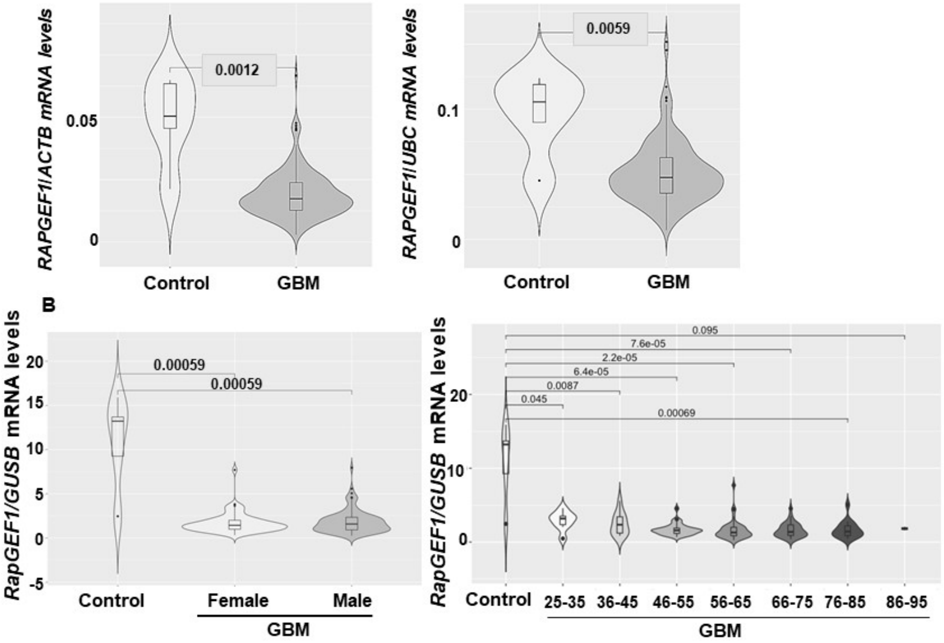

Supplementary Figure 1–Analysis of C3G expression in glioblastoma patient samples. (A) RapGEF1 mRNA levels in glioblastoma patient samples normalized with different housekeeping genes: left panel, *ACTB* (β-actin) and right panel, *UBC* (ubiquitin C). **(B)** *RAPGEF1* mRNA levels in GBM patient samples grouped by sex (left panel) or age (right panel).

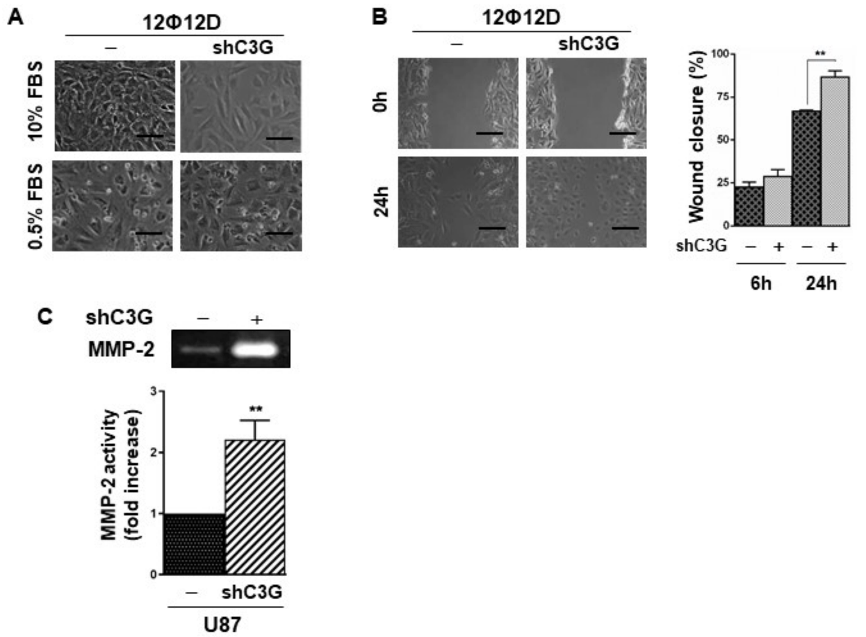

Supplementary Figure 2–C3G down-regulation enhances migration of 12Φ12D glioblastoma cells. Lack of effect of non-targeting shRNAs on invasive properties of GB U87 cells. (A) Representative phase contrast microscopy images of 12Φ12D cells morphology. Scale bars: 50 μm. **(B)** Wound healing assay in 12Φ12D cells. Left panel, representative phase contrast microscopy images at 0h and 24h of migration; right panel, histogram represents the mean value of wound closure percentage ± S.E.M. at 6 and 24h (n=3-4). **p≤0.01 C3G silenced cells versus non-silenced. Scale bars: 100 μm. **(C)** Zymographic analysis of MMP2 activity of non-silenced and C3G silenced U87 cells using culture medium from 24h serum-deprived cells that is submitted to electrophoresis in 8% SDS-polyacrylamide-0.1% gelatin gels under non-reducing conditions[1]. Upper panel, representative zymogram; lower panel, histogram showing the fold increase of MMP-2 activity (mean value ± S.E.M., n=5), p≤0.01, compared to non-silenced cells.

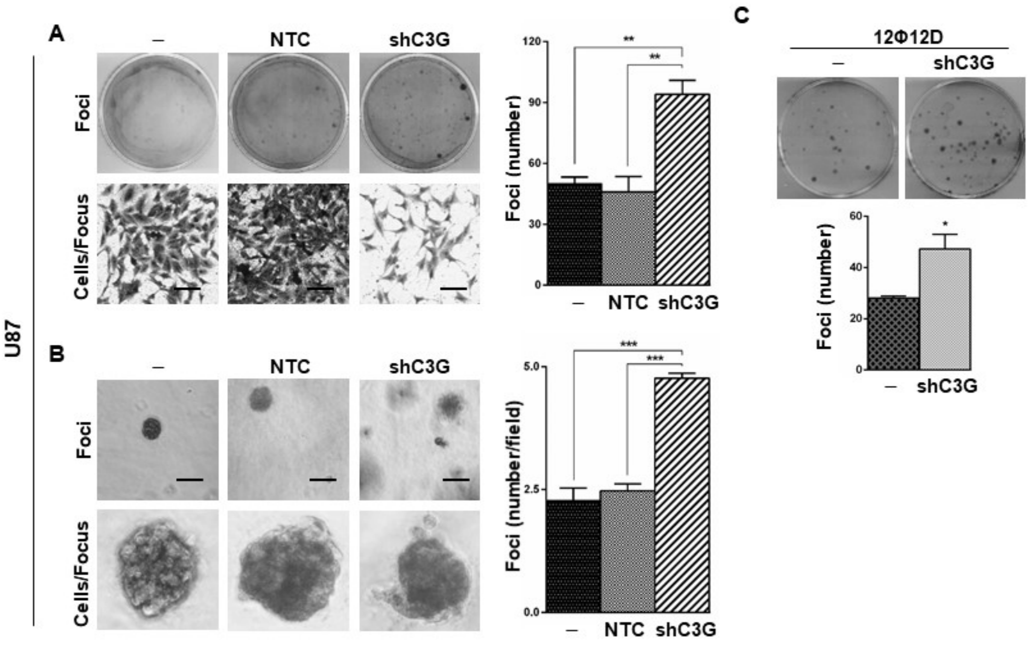

Supplementary Figure 3–Non-targeting shRNAs does not interfere with tumorigenic properties of glioblastoma cells. (A) and (B) Effect of non-targeting shRNAs (NTC) as compared to parental and C3G silenced U87 cells. **(A)** Anchorage-dependent growth. Left panels, representative images of foci macroscopic view (upper panel) and cells within an individual focus (lower panel); right panel, histogram showing the mean value ± S.E.M. of foci number (n=3). Scale bars: 100 μm. **(B)** Anchorage-independent growth. Left panel, representative images of foci microscopic view (upper panel) and cell organization within an individual focus (lower panel); right panel, histogram showing the mean value ± S.E.M. of foci number (n=3). Scale bars: 100 μm. **(C)** Effect of C3G silencing on anchorage-dependent growth of 12Φ12D cells. Upper panel, representative images of foci macroscopic view; lower panel, histogram showing the mean value ± S.E.M. of foci number (n=3). *$p \leq 0.05$, **$p \leq 0.01$, ***$p \leq 0.001$, compared as indicated.

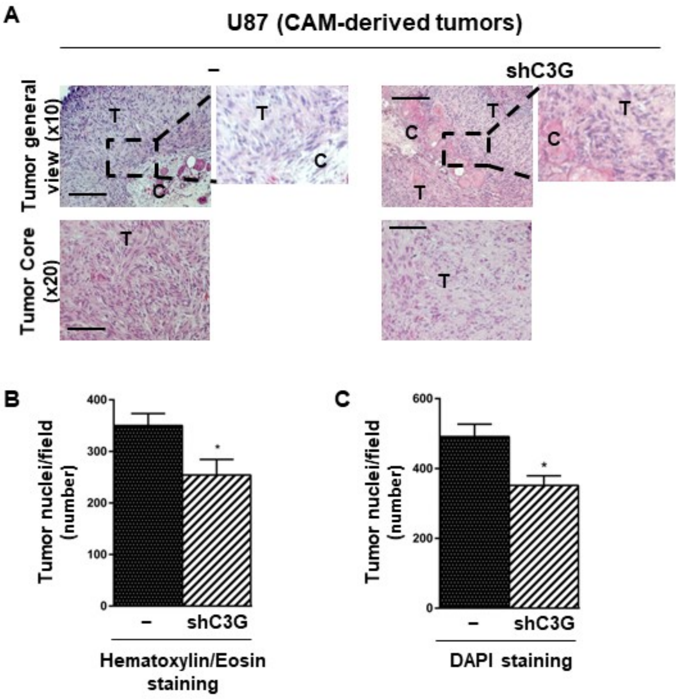

Supplementary Figure 4–Analysis of the morphology and cell density of tumors generated by parental and C3G-silenced U87 cells in CAM assays. (A) Hematoxylin/eosin staining of tumor sections. Phase contrast microscopy images. Upper panel: left side, general view at 10x magnification, where the tumor (T) and the CAM tissue (C) can be visualized; right side, magnification of the rectangle area. Lower panel, view of tumor core at 20x magnification. Scale bars: 100 μm (upper panel) and Scale bars: 50 μm (lower panel). **(B and C)** Histograms showing the mean value ± S.E.M. of the number of tumor cells or nuclei per field from CAM derived tumors stained with hematoxylin/eosin **(B)** or with anti-αSMA **(C)** (n=3). *$p \leq 0.05$ compared as to tumors derived from non-silenced cells.

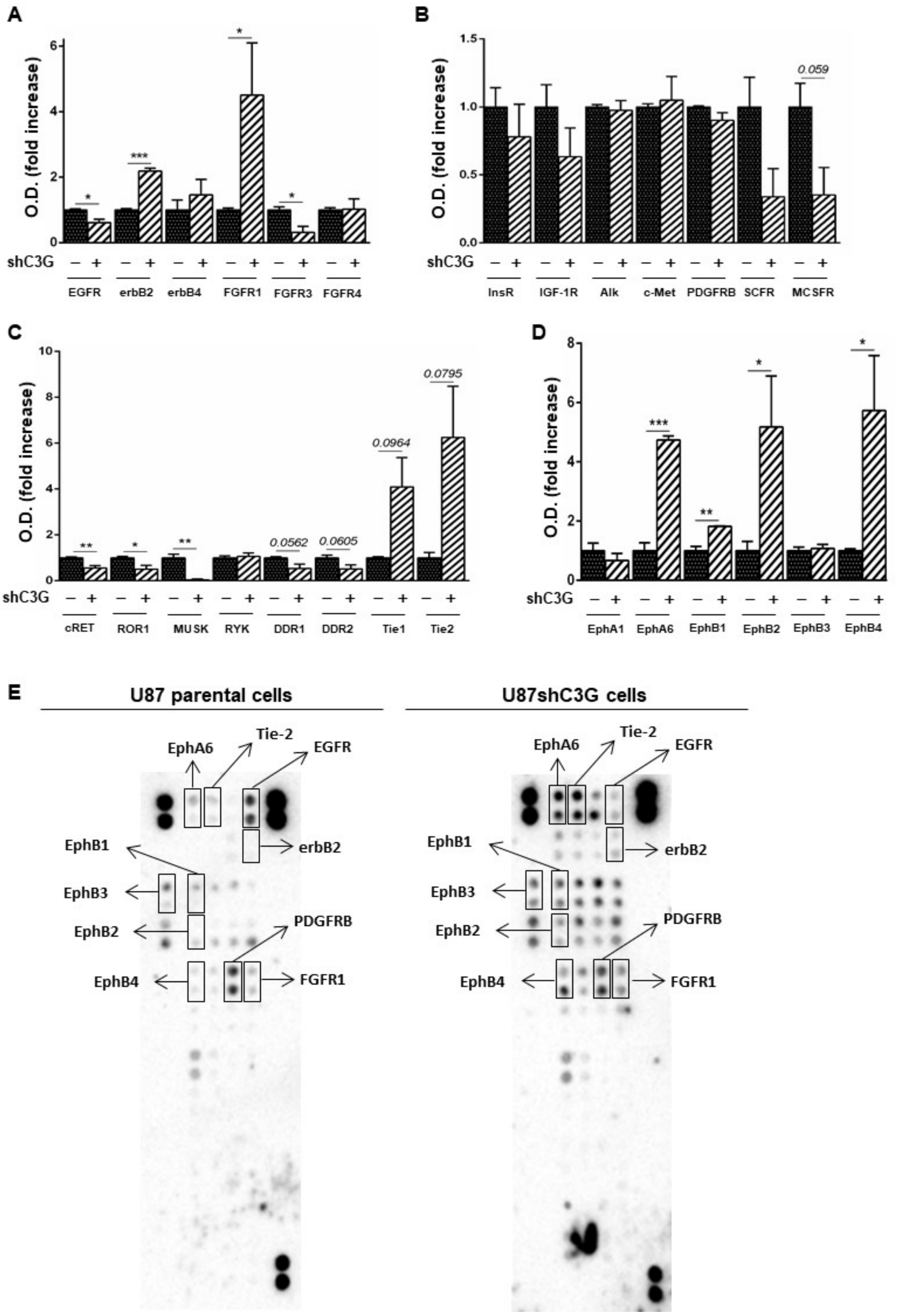

Supplementary Figure 5–Effect of C3G silencing on phosphorylation of RTKs using an array. (A, B, C and D) Histograms represent the mean value ± S.E.M. of the densitometric quantification of the phosphorylation levels of RTKs in parental and C3G silenced U87 cells (n=2). **(E)** Representative images of the membranes, where some RTK phosphorylation spots are indicated. *p≤0.05, **p≤0.01, ***p≤0.001 compared as indicated.

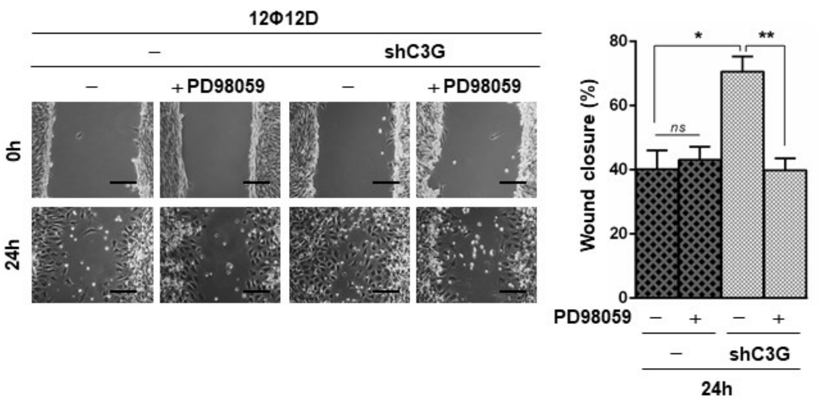

Supplementary Figure 6-ERKs inhibition prevents the increase in migration induced by C3G silencing in 12Φ12D glioblastoma cells. Wound healing assay in 12Φ12D cells maintained untreated or treated with PD98059. Left panels, representative phase contrast microscopy images at 0h and 24h of migration; right panel, histogram represents the mean value of wound closure percentage ± S.E.M. at 24h (n=3-4). *p≤0.05, **p≤0.01, compared as indicated. Scale bars: 100 μm.

References

1. Priego N, Arechederra M, Sequera C, Bragado P, Vazquez-Carballo A, Gutierrez-Uzquiza A*, et al.* C3G knock-down enhances migration and invasion by increasing Rap1-mediated p38alpha activation, while it impairs tumor growth through p38alpha-independent mechanisms. *Oncotarget* 2016, **7**(29)**:** 45060-45078.
